# Murine Leukemia Virus Uses TREX Components for Efficient Nuclear Export of Unspliced Viral Transcripts

**DOI:** 10.3390/v6031135

**Published:** 2014-03-10

**Authors:** Toshie Sakuma, Jason M. Tonne, Yasuhiro Ikeda

**Affiliations:** Department of Molecular Medicine, Mayo Clinic, Rochester, MN 55905 USA; E-Mails: tonne.jason@mayo.edu (J.M.T.); ikeda.yasuhiro@mayo.edu (Y.I.)

**Keywords:** gammaretrovirus, MLV, UAP56, THOC5, THOC7, TREX, RNA nuclear export

## Abstract

Previously we reported that nuclear export of both unspliced and spliced murine leukemia virus (MLV) transcripts depends on the nuclear export factor (NXF1) pathway. Although the mRNA export complex TREX, which contains Aly/REF, UAP56, and the THO complex, is involved in the NXF1-mediated nuclear export of cellular mRNAs, its contribution to the export of MLV mRNA transcripts remains poorly understood. Here, we studied the involvement of TREX components in the export of MLV transcripts. Depletion of UAP56, but not Aly/REF, reduced the level of both unspliced and spliced viral transcripts in the cytoplasm. Interestingly, depletion of THO components, including THOC5 and THOC7, affected only unspliced viral transcripts in the cytoplasm. Moreover, the RNA immunoprecipitation assay showed that only the unspliced viral transcript interacted with THOC5. These results imply that MLV requires UAP56, THOC5 and THOC7, in addition to NXF1, for nuclear export of viral transcripts. Given that naturally intronless mRNAs, but not bulk mRNAs, require THOC5 for nuclear export, it is plausible that THOC5 plays a key role in the export of unspliced MLV transcripts.

## 1. Introduction

Nucleocytoplasmic transport of mRNA is regulated by RNA-binding proteins. Two major classes of transport receptors are known to mediate viral RNA export: nuclear export factor 1 (NXF1) and karyopherins, also known as importins/exportins [1]. While karyopherins involve GTPase Ran to deliver RNA cargo, NXF1 requires several adaptor proteins to export viral RNAs [1,2]. Most retroviruses, including human immunodeficiency virus type 1 (HIV-1), mouse mammary tumor virus, and human T cell leukemia virus encode viral proteins Rev, Rem and Rex, respectively. These viral proteins interact within a *cis*-element in the viral genome, and promote nuclear export of intron-containing viral RNAs through a karyopherin, CRM1 (chromosome region maintenance 1), also known as XPO1 (exportin1) [3,4,5,6,7,8,9,10,11]. In addition to CRM1-dependent unspliced retroviral RNA export, unspliced viral transcripts of simple retroviruses, such as Mason-Pfizer monkey virus (MPMV), use NXF1, which is implicated in bulk mRNA nuclear export [2,12]. The constitutive transport element (CTE) of MPMV directly interacts with NXF1 and prevents the nuclear retention of unspliced viral transcripts in the nucleus [2,13]. We have reported that gammaretrovirus, murine leukemia virus (MLV) utilizes NXF1 for export of unspliced viral transcripts [14]. However, the mechanism of unspliced viral RNA export from the nucleus to the cytoplasm is not fully understood. 

Successful RNA processing requires recruitment of multiple nuclear proteins on bulk mRNAs [15,16]. Eukaryotic mRNA production is initiated by RNA splicing of pre-mRNA and is regulated by multiple nuclear proteins prior to interaction with NXF1 in order to reach the nuclear pore complex (NPC) [17]. The transcription export (TREX) complex has a major role in bulk mRNA export [18,19] as well as intronless mRNAs [20,21,22]. Known components of the human TREX complex are UAP56 [18,23,24], Aly/REF [25], UIF [26], GANP [27], CHTOP [28], CIP29 [29], and THO [30]. A TREX component of RNA helicase UAP56 is first recruited to the mRNA along with the adaptor protein Aly/REF [31,32,33]. These messenger ribonucleoprotein (mRNP) complex formations (TREX-mRNAs) provide a license for mRNA to be exported via the NXF1 pathway [34]. Interaction of viral proteins with the TREX components also plays an important role for exporting the intronless viral transcripts of herpesviruses and avian influenza virus [35,36,37].

In this study, we examined whether TREX components are involved in MLV RNA export. Depletion of TREX components by siRNAs demonstrated that UAP56 is required for efficient nuclear export of both unspliced and spliced viral transcripts. Under a limited loss of function by targeting THO complex by siRNAs [38], we also determined that THOC5 and THOC7 are required for efficient nuclear export of unspliced viral RNAs. 

## 2. Results and Discussion

### 2.1. Involvement of UAP56 in the Nuclear Export of MLV Transcripts

**Figure 1:**
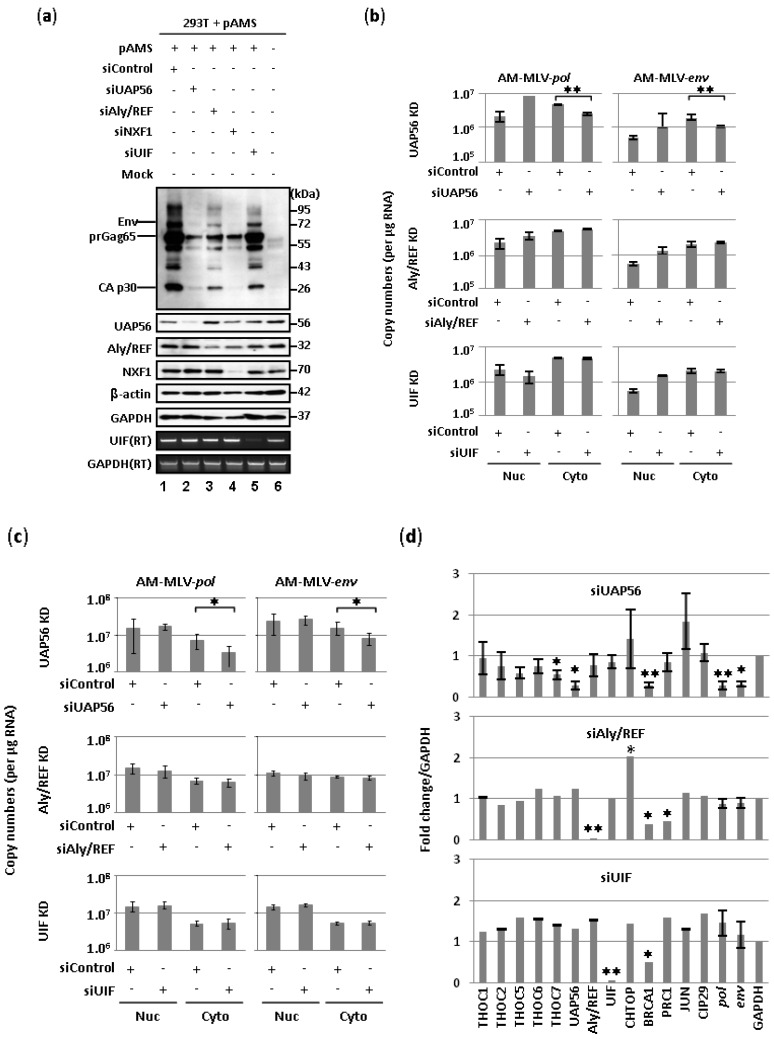
UAP56 is involved in the nuclear export of murine leukemia virus (MLV) transcripts. (**a**) Influence of disruption of adaptor proteins, UAP56, Aly/REF and UIF, on the expression of AM-MLV proteins in 293T cells transfected with pAMS. 293T cells were transfected with the indicated siRNA, and then transfected with 0.4 µg of pAMS at 24 h post-siRNA transfection. At 30 h post-transfection of pAMS, cells were harvested for protein analysis; (**b**) RT-qPCR analysis of unspliced (AM-MLV-*pol*) and spliced (AM-MLV-*env*) transcripts. AM-MLV-infected 293T cells were treated with the indicated siRNAs, and averages of viral RNA copy numbers from three independent experiments were determined by RT-qPCR. Error bars represent standard deviation with two-tailed Student’s *t*-test (** *p* < 0.01); (**c**) RT-qPCR analysis of unspliced (AM-MLV-*pol*) and spliced (AM-MLV-*env*) transcripts. AM-MLV-infected TE671 cells were treated with the indicated siRNAs, and averages of viral RNA copy numbers from three independent experiments were determined by RT-qPCR. Error bars represent standard deviation with two-tailed Student’s *t*-test (* *p* < 0.05); (**d**) Normalization of RT-qPCR results. AM-MLV-infected TE671 cells were treated with the indicated siRNAs, and quantitative gene expression was normalized to GAPDH. Cytoplasmic fractions of RNA were used for the analysis (*n* = 3). Error bars represent standard deviation with two-tailed Student’s *t*-test (* *p* < 0.05, ** *p* < 0.01).

Recruitment of NXF1 to mRNA requires TREX components in metazoans [30]. We have previously shown that NXF1 is involved in MLV mRNA export [14]. To assess the involvement of TREX components in the nuclear export of MLV transcripts, we first examined whether disruption of major components of TREX, UAP56 and Aly/REF, affects the expression of MLV proteins (Figure 1). Transfection of the UAP56-specific siRNA resulted in the reduction of viral protein expression (Figure 1a, lane 2) as similar to the expression observed in NXF1-depleted cells (Figure 1a, lane 4). The depletion of Aly/REF also showed a slight reduction of viral protein (Figure 1a, lane 3). We also examined another adaptor protein, UAP56-interacting factor (UIF) [26]. UIF is recruited to the mRNA via a direct interaction with the histone chaperone FACT, together with Aly/REF to ensure the efficient mRNA export through NXF1 [26]. UIF also facilitates delivery of the intronless Kaposi’s sarcoma-associated herpesvirus (KSHV) mRNAs to the nuclear pore via NXF1-dependent pathway [39]. In case of MLV viral export, however, depletion of UIF did not affect the expression of viral proteins (Figure 1a, lane 5). We then assessed the nuclear and cytoplasmic viral RNA levels upon depletion of UAP56, Aly/REF or UIF (Figure 1b). Significant reduction of unspliced and spliced viral RNAs in the cytoplasm was observed when MLV-infected 293T cells were treated with the UAP56-targeted siRNA. Depletion of Aly/REF or UIF did not affect the level of viral transcripts in the nucleus or the cytoplasm. The same experiment was conducted by using MLV-infected TE671 cells (Figure 1c). Similar to the results from MLV-infected 293T cells, knockdown of UAP56 significantly reduced the unspliced viral RNA levels in the cytoplasm (Figure 1c). Because UAP56 has been reported to affect transcription [18] and mitosis [40], we also examined whether the knockdown of UAP56, Aly/REF, or UIF has an effect on global mRNA expression (Figure 1d). BRCA1 and PRC1 were used as controls for UAP56 dependent and independent genes, respectively [40]. Knockdown of UAP56 significantly reduced the level of BRCA1 (Figure 1d). Other genes tested were not affected by depletion of UAP56 as reported [41], suggesting depletion of UAP56 specifically down regulated MLV RNA. Our data thus imply that MLV uses UAP56 and NXF1 for cytoplasmic accumulation of viral transcripts. 

### 2.2. Depletion of THO Complex Prevents the Cytoplasmic Accumulation of Unspliced MLV Transcripts

Components of TREX complex are formed by assembly of UAP56, adaptor proteins, and THO complex, which include THOC1, THOC2, THOC3, THOC5, THOC6, and THOC7 [42,43]. Association of mRNA with the THO/TREX complex is required to recruit NXF1 export receptor before nuclear export of mRNAs [18,44]. Based on different classes of RNA transcripts, mRNAs recruit different components of nuclear proteins for nuclear export. In addition to UAP56 and Aly/REF as TREX components, recent findings advanced the field of the study by identifying new components of TREX complex [29,32]. Although mRNAs still use NXF1 at the last stage of the RNA export, a recruitment of different adaptor proteins in TREX components are distinguished from TREX, thus named as alternative mRNA export (ALREX), or TREX2 [27,45,46].

**Figure 2:**
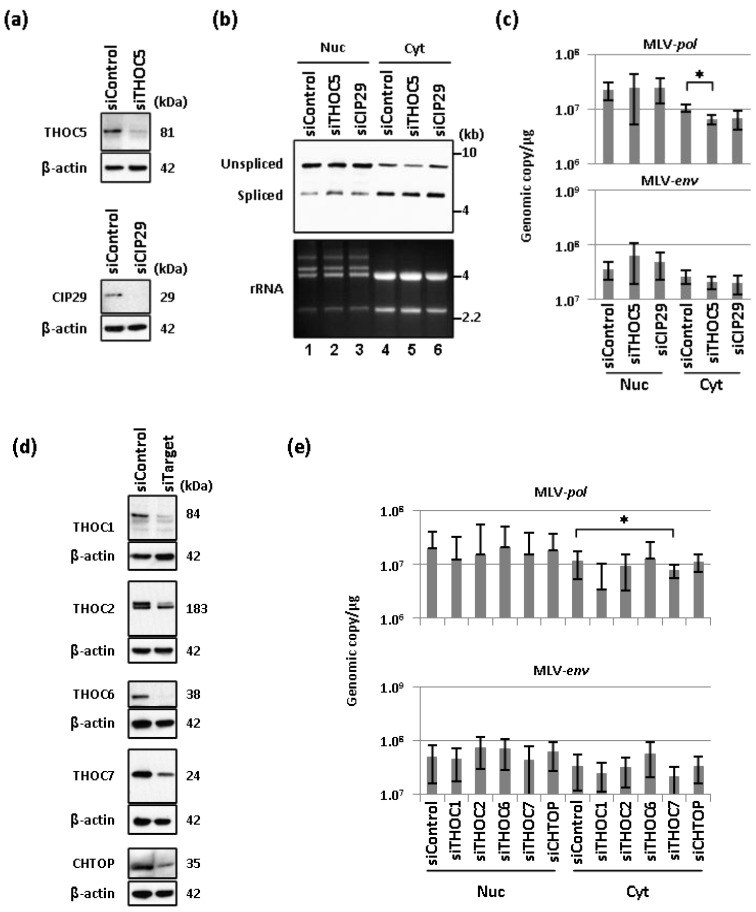
THOC5 and THOC7 are essential for MLV RNA export. (**a**) Western blotting was performed to access siRNA-induced knockdown. Protein size is indicated in kDa. β-actin is stained as a loading control; (**b**) Northern blot analysis was performed to examine the effect of siRNA targeting indicated genes in the AM-MLV-infected TE671 cells. Nuclear and cytoplasmic RNA fractions were harvested at 48 h after transfection. RNA size in kb was indicated; (**c**) RT-qPCR was performed to quantify viral transcripts in the nucleus and the cytoplasm upon siRNA treatment (siControl, siTHOC5 or siCIP29) in AM-MLV-infected TE671 cells. An average of unspliced and spliced viral RNA copies (per μg of RNA) from three independent experiments is shown. Error bars indicate standard deviation with two-tailed Student’s *t*-test (* *p* < 0.05); (**d**) Western blotting was performed to confirm siRNA-induced knockdown effects. Protein size is indicated in kDa. β-actin is stained as a loading control; (**e**) RT-qPCR was performed to quantify viral transcripts in the nucleus and the cytoplasm upon siRNA treatment in AM-MLV-infected TE671 cells. Viral RNA copy (per μg of RNA) was compared to the cells treated with control siRNA and cells treated with siRNA targeting THOC1, THOC2, THOC6, THOC7, or CHTOP. An average of three independent experiments is summarized. Error bars indicate standard deviation with two-tailed Student’s *t*-test (* *p* < 0.05).

Our data showed UAP56 plays a key role for the MLV nuclear export of unspliced viral transcripts (Figure 1). Based on this, we next asked if nuclear proteins other than UAP56 make a distinction between unspliced and spliced viral transcripts during RNA processing. For this purpose, we examined the influence of disruption of components of THO complex by siRNA on nuclear export of MLV transcripts. THOC5 is a co-adaptor of TREX component, and interacts with NXF1 directly [47]. Because studies have shown that UAP56 can directly interact with one of the TREX components CIP29 [29] and THO complex [31], we first examined knockdown effects of CIP29 and THOC5 in cells persistently infected with MLV. Western blot analysis verified the siRNA-mediated depletion of THOC5 or CIP29 (Figure 2a). Representative northern blotting data from three independent experiments showed that depletion of THOC5 reduced the cytoplasmic levels of unspliced viral transcripts (Figure 2b, lane 2 and 5). Quantitative RT-qPCR analysis of THOC5 depleted cells also showed the significant reduction of unspliced viral transcripts (MLV-*pol*) in the cytoplasm (Figure 2c). No significant change in the levels of spliced viral transcripts (MLV-*env*) was observed upon disruption of THOC5 (Figure 2c). Although depletion of CIP29 also showed a trend of reduced viral transcripts in the cytoplasm, these changes were not significant due to variations between experiments. Thus, our data suggests the involvement of THOC5 in MLV nuclear export.

Although naturally intronless *HSP70* mRNA recruits THOC5 to allow direct interaction with the NTF2-like domain of NXF1, THOC5 is not essential for the export of bulk mRNA [47]. Our data showed that depletion of THOC5 did not affect the level of spliced viral transcript and only reduced the cytoplasmic accumulation of unspliced viral RNA. Because MLV nuclear export does not require Aly/REF (Figure 1), it is possible that THOC5 is a major adaptor protein to recruit NXF1 for the unspliced viral RNA. Viral protein ORF57 in KSHV also recruits THOC5 onto the intronless viral mRNA so that host cellular machinery can recognize the intronless viral mRNAs as a spliced viral mRNAs [35]. Unlike complex retroviruses or KSHV, MLV does not encode accessory proteins to mediate the nuclear export of viral RNA. Thus, similar to those on intronless mRNAs [21,22], a recruitment of THOC5 on unspliced MLV transcripts may be controlled by viral sequences. 

Since MLV nuclear export requires a component of THO complex, THOC5 (Figure 2a–c), we further examined whether MLV nuclear export requires subunits of THO complex, including THOC1, THOC2, THOC6, and THOC7. We verified the knockdown effects of siRNA targeting each THO subunit by Western blot (Figure 2d). Quantitative RT-qPCR analysis was performed to determine the influence of THO on the nuclear and cytoplasmic levels of spliced and unspliced MLV transcripts (Figure 2e). Depletion of THOC7 showed statistically significant reduction in unspliced viral transcripts (MLV-*pol*) in the cytoplasm (Figure 2e, top). Depletion of other THO subunits, *i.e.*, THOC2, and THOC6, did not affect the viral RNA levels in the cytoplasm although a modest reduction of the unspliced viral RNA by siRNA targeting THOC1 was observed in the cytoplasm. Depletion of THO subunits did not lead to significant changes in the levels of spliced MLV-*env* transcripts (Figure 2e, bottom). Recently, Chang *et al.* reported a new component of TREX, CHTOP, which binds and activates the activity of UAP56, and binds NTF2-like domain of NXF1 by competing with THOC5 [28]. In addition to the THO components, we also examined the involvement of CHTOP. As shown in Figure 2d,e, however, depletion of CHTOP did not affect the viral RNA levels, suggesting that CHTOP is not critical for the nuclear export of MLV transcripts. Our data therefore demonstrate the critical roles of UAP56, two subunits of THO complex (THOC5 and THOC7) and NXF1 [14] in MLV nuclear export.

**Figure 3:**
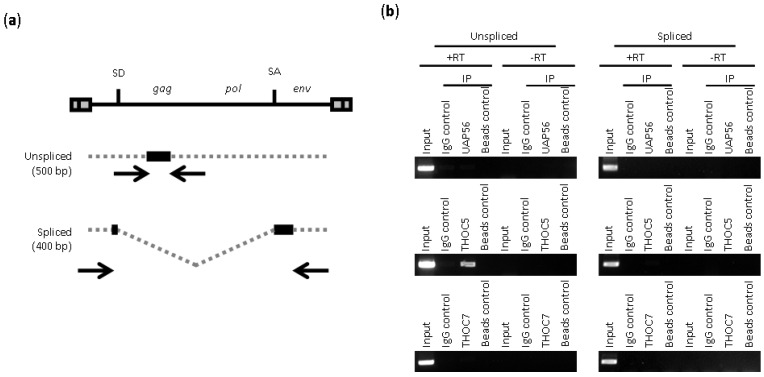
THOC5 interacts with unspliced MLV transcript. (**a**) Schematic representation of MLV genomic RNA and viral mRNAs. Arrows indicate the positions and the orientation of the PCR primers for RIP assay. The black square indicates the expected PCR products with predicted size at the left; (**b**) RIP assay was performed to detect UAP56-, THOC5-, or THOC7-associated viral transcripts. Immunoprecipitation (IP) was conducted in the presence of isotype control (IgG control), UAP56, THOC5, or THOC7 antibodies. IP without any antibodies (Beads control) is also included as a negative control. RT-PCR was performed to detect MLV transcripts in RNA samples isolated from the immunoprecipitates with (+RT) or without (−RT) reverse transcriptase.

In order to examine the interaction of the viral transcripts and components of TREX complex (*i.e.*, UAP56, THOC5, and THOC7), we performed RNA immunoprecipitation assay. Regions of primers targeting unspliced and spliced MLV genome are summarized in Figure 3a. RT-PCR analysis showed that only unspliced viral transcript interacted with THOC5 (Figure 3b). Although UAP56 is involved in the unspliced and spliced viral transcripts, our result did not support the direct interaction of UAP56 to the viral transcript. Similarly, the interaction of THOC7 to viral transcript was absent. These results suggest that THOC5 is a component of RNA-binding complex and an essential component for nuclear export of unspliced MLV. We speculate that UAP56 and THOC7 are recruited to viral transcripts through an adaptor protein, such as THOC5 in the case of unspliced viral transcripts. 

**Figure 4:**
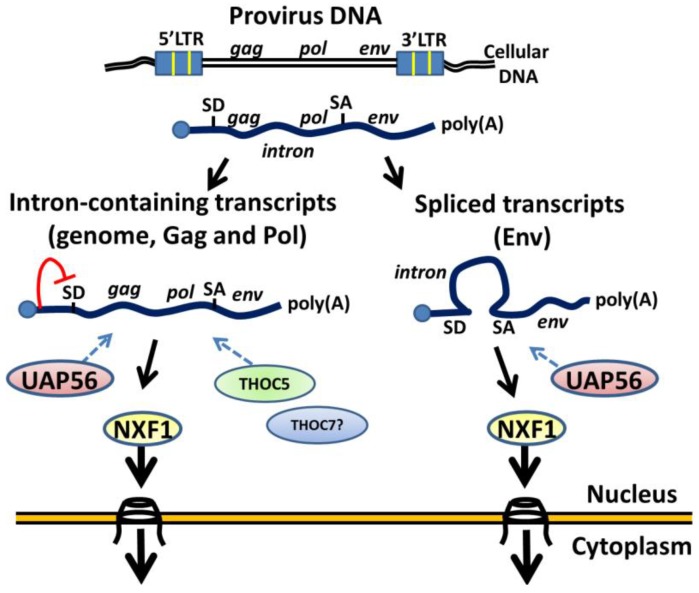
A model of gammaretroviral RNA export. Both unspliced and spliced transcripts require UAP56 and NXF1. THOC5 and THOC7 are required for the nuclear export unspliced viral transcripts. SD: splice donor; SA: splice acceptor.

In this study, we screened the essential factors for MLV nuclear export by using siRNAs. We have shown that depletion of TREX components, UAP56, THOC5 and THOC7, impairs the cytoplasmic levels of viral transcript. Other simple retroviruses require only NXF1 via *cis*-element to allow unspliced viral transcripts to leave the nucleus [2,13]. Our results therefore indicate that, unlike other simple retroviruses with CTEs, MLV recruits TREX components for nuclear export of unspliced viral transcripts via the NXF1 pathway (Figure 4). Further studies will unveil whether these proteins recognize the viral sequence in a sequence-specific manner.

## 3. Experimental Section

### 3.1. Cell Lines and Transfection

293T and TE671 cells were cultured in Dulbecco’s modified Eagle’s medium with 10% fetal bovine serum at 37 °C with 5% CO_2_. Persistent AM-MLV-infected TE671 cells were generated as described previously [14]. Similarly, 293T cells chronically infected with MLV were generated by transfecting 293T cells with pAMS (ATCC). After passaging the cells for three times, viral protein production was confirmed by Western blot. For siRNA transfection, Dharma*FECT* transfection reagent (Dharmacon, Pittsburgh, PA, USA) was used according to the manufacture’s instruction. ON-TARGETplus Non-targeting siRNA (Thermo Scientific, Rockford, IL, USA) was used for siRNA control. siRNAs targeting NXF1, UAP56, THOC1, THOC2, THOC5, THOC6, THOC7, and CHTOP were purchased from Qiagen (Gaithersburg, MD, USA). siRNAs targeting Aly/REF, and UIF were purchased from ThermoScientific. For Western blot analysis, 293T cells (2 × 10^5^ cells/well) were plated in a 6 well plate 24 h prior to siRNA transfection (25 nM), and 1.0 µg of plasmid was transfected with FuGENE6 (Promega, Madison, WI, USA) 24 h after siRNA transfection, and cells were then harvested at 48 h after plasmid transfection. For cell lines chronically infected with MLV, cells were transfected with 25 nM of siRNAs, and harvested at 48 h post-transfection.

### 3.2. Northern Blotting

Probes were generated by using North2South Random Prime DNA Biotinylation Kit (Pierce, Rockford, IL, USA) as described previously [14]. Two micrograms of cytoplasmic and nuclear RNA samples were used for the analysis. RNAs were heated at 65 °C for 10 min before loading on 1.2% formaldehyde agarose gel. As a loading control, the ethidium bromide-staining pattern of nuclear (45S, 32S and 20S) and cytoplasmic (28S and 18S) ribosomal RNA were visualized under UV light. Probe hybridization and signal detection was performed by following a protocol provided by the North2South Chemiluminescent hybridization and Detection Kit (Pierce).

### 3.3. RT-PCR/RT-qPCR

Nuclear and cytoplasmic RNA samples were extracted by using a PARIS kit (Ambion, Carlsbad, CA, USA) according to the manufacturer’s protocol. Total RNA was extracted by RNeasy Plus Mini Kit (Qiagen, Gaithersburg, MD, USA). The RNA concentrations were determined by a NanoDrop Spectrophotometer (Thermo Scientific) and 1.0 µg of RNA was used for cDNA synthesis (RNA-to-cDNA EcoDry Premix, Clontech, Mountain View, CA, USA). Primers for UIF were 5'-ATGAACCGGTTTGGTACCCGG-3' and 5'-CTATCCCACGGTGACAAAGCG-3', and GAPDH was amplified by 5'-GTCCATGCCATCACTGCCA-3' and 5'-TTACTCCTTGGAGGCCAT-3'. The real-time qPCR assay was performed as described previously [48,49]. For global gene expression analysis, all the probes and primers were purchased from IDT (Coralville, IA, USA). 

### 3.4. Western Blot

Protein samples were first lysed in RIPA buffer. Cell debris was centrifuged and the supernatants were mixed with Laemmli sample buffer, supplemented with β-mercapto, USAthanol. The samples were boiled at 95 °C for 5 min and subjected to 4%–15% SDS-PAGE (Bio-Rad, Hercules, CA), and transferred to a polyvinylidene diflouride membrane. Membranes were blocked in 5% milk/PBS-0.2% Tween 20 and probed with the indicated antibodies. BAT1 polyclonal antibody was purchased from Abnova. Mouse anti-Aly/REF, clone 11G5 was purchased from Millipore. Rabbit anti-NXF1 antibody was purchased from Sigma. Goat-anti MLV antibody was purchased from ATCC. Polyclonal antibodies for CIP29 and THOC5 were purchased from Pierce. Mouse HRP-1 affinity purified polyclonal antibody was purchased from R&D Systems. Polyclonal antibodies for THOC2, THOC6, THOC7, CHTOP were purchased from Proteintech Group. 

### 3.5. RIP (RNA Immunoprecipitation) Assay

RIP assay was performed as described previously [14]. Briefly, nuclear lysate was isolated from TE671 cells infected with MLV by using buffers supplied by PARIS kit (Ambion, Carlsbad, CA, USA). After shearing, cells were pelleted at 2,000× *g* for 5 min at 4 °C. Two milligrams of the supernatant and 1 µg of antibody were incubated for 2 h at 4 °C. Protein A Sepharose CL-4B beads (GE Healthcare, Little Chalfont, Buckinghamshire, UK) were added to the mixture, and incubated for 1 h at 4 °C. Beads were washed with RIP buffer for five times, followed by one wash in PBS. RNA was extracted by RNeasy Plus mini kit (Qiagen), and cDNA was synthesized (Clontech) for PCR reaction. Unspliced viral transcript, AM-MLV *gag*, was amplified by using primers 5'-ATGGGCCAGACTGTTACCACT-3' and 5'-TGGTCTTGGGTCCCTATAAGGC-3'. Spliced viral transcript, AM-MLV *env*, was amplified by using primers 5'-ATCGGGAGACCCCTGCCCAGGGACCA-3' and 5'-TCTCCAGGTTACATTAAAGACCTGATGG-3'.

## 4. Conclusions

In this study, we have studied the RNA export mechanism of MLV and found: (i) cellular factor UAP56 is involved in the nuclear export of both spliced and unspliced viral RNAs; and (ii) the recruitment of THO component is essential for the export of unspliced RNAs. Because recruitment of TREX complex is utilized by other human pathogens, such as herpesviruses, our study provides novel insight into the host-virus interaction within the nuclear export of a MLV life cycle.
